# High-sensitivity troponin-T levels and associated health conditions in 3146 women aged 46

**DOI:** 10.1080/07853890.2023.2264340

**Published:** 2023-10-05

**Authors:** Meri-Maija Ollila, Riikka K. Arffman, Kari Kaikkonen, Laure Morin-Papunen, Juhani Junttila, Terhi T. Piltonen

**Affiliations:** a Department of Obstetrics and Gynaecology, Medical Research Center Oulu, Research Unit of Clinical Medicine, University of Oulu and Oulu University Hospital, Oulu, Finland; b Research Unit of Internal Medicine, Medical Research Center Oulu, University of Oulu and Oulu University Hospital, Oulu, Finland

**Keywords:** Troponin, oligomenorrhea, polycystic ovary syndrome, hyperandrogenism, menopause

## Abstract

**Objective:**

The aim of the study was to investigate are there associations between common female sex-specific health conditions (oligo/amenorrhea, hyperandrogenism, menopause and polycystic ovary syndrome [PCOS]) and high-sensitivity troponin-T (hs-TnT) levels.

**Methods:**

Cross-sectional and longitudinal analyses of a general population-based prospective cohort study were performed. The hs-TnT levels of 3146 women aged 46 were measured using an Elecsys® Troponin T high-sensitivity assay. Median hs-TnT levels and 25 and 75 percentiles of the cases and controls were compared. Also, a logistic regression analysis using a binary outcome – undetectable hs-TnT (< 3.0 ng/L) versus detectable hs-TnT (≥ 3.0 ng/L) – was performed.

**Results:**

Women with oligo/amenorrhea at age 31 had significantly higher hs-TnT levels at age 46 than women without oligo/amenorrhea (4.06 [3.59; 4.86] vs 3.98 [3.44; 4.71] ng/L, *p* = .042). Menopausal women had significantly higher hs-TnT levels than premenopausal women (4.15 [3.54; 4.91] vs 3.95 [3.45; 4.68] ng/L, *p* = .012) at age 46. Women with PCOS or hyperandrogenism had comparable hs-TnT levels with their controls. In the adjusted logistic regression analysis, oligo/amenorrhea (odds ratio [OR] = 1.52 [0.90–2.57]), hyperandrogenism (OR = 1.20 [0.75–1.92]), PCOS (OR = 1.51 [0.81–2.84]) and menopause (OR = 1.05 [0.63–1.74]) were not significantly associated with detectable hs-TnT.

**Conclusions:**

This study was the first to investigate how oligo/amenorrhea, hyperandrogenism, PCOS and menopause are associated with hs-TnT. Although women with oligo/amenorrhea and menopause had higher hs-TnT levels than women without these conditions, the difference was small. Larger studies are required to better understand the effects of oligo/amenorrhea on cardiovascular health.

## KEY MESSAGES

No previous studies have investigated the association between common female sex-specific health conditions, such as oligo/amenorrhea, hyperandrogenism and PCOS, and hs-TnT levels. Only one prior study has investigated the association between menopause and hs-TnT levels.

Hs-TnT levels were significantly higher in women with oligo/amenorrhea and relatively early menopause at age 46 than women without these conditions, whereas women with hyperandrogenism or PCOS and their controls have comparable hs-TnT levels.

The effect of oligo/amenorrhea on cardiovascular health should be further investigated. A simple question about the presence of oligo/amenorrhea might identify women at increased risk of cardiovascular disease.

## Introduction

Troponin (Tn) is a complex of three regulatory proteins, troponin-C, -I and -T, and is involved in the contraction of skeletal and cardiac muscles. Increased levels of high-sensitivity cardiac troponin (hs-Tn) are used as biomarkers of myocardium damage in everyday clinical practice. Moreover, even a minimal increase in hs-Tn within the normal range – that is, below the 99th percentile – may be a predictor of cardiovascular disease (CVD) and mortality in the asymptomatic general population [1]. Cardiac troponins can also improve CVD risk prediction when added to traditional CVD risk factors or risk score calculators; meaning that hs-Tn can be used to evaluate residual CVD risk that is not covered by traditional risk factors such as hypertension or dyslipidaemia [1].

High-sensitivity assays have shown that women have lower levels of cardiac troponin than men, which might be because the left ventricular mass is lower in women [2]. During a woman’s lifespan, the risk of CVD is modified by female-specific health conditions, especially menopause. The presence of common health concerns, such as oligo/amenorrhea and polycystic ovary syndrome (PCOS), during the reproductive years, has been associated with an increased risk of CVD events and mortality [3, 4]. After menopause, low oestrogen levels and a relative increase in androgen levels significantly promote CVD event risk [5]. However, there is a lack of literature on how these common female sex-specific health conditions affect troponin levels. To date, only one study has investigated the effect of menopause on hs-Tn levels [6] showing that high-sensitivity troponin-T (hs-TnT) is not associated with menopause, and no studies have focused on oligo/amenorrhea, hyperandrogenism, or PCOS. This information would be needed both in clinical practice when evaluating hs-Tn levels but also in the research field, when optimizing the use of hs-Tn as a risk prediction tool.

Therefore, the objective of the present study was to investigate whether there is any association between common female sex-specific health conditions (oligo/amenorrhea, hyperandrogenism, PCOS and menopause) and hs-TnT levels in a large population-based setting.

## Materials and methods

### Study population

This study is based on a general population-based prospective Northern Finland Birth Cohort 1966 (NFBC1966). The birth cohort includes all children (12,231 total of whom 5889 are female) who were born in the two northernmost provinces of Finland in 1966. The original study evaluated the early life factors of long-term health and work ability. Since the beginning, the cohort population has been followed at four different time points: 1, 14, 31 and 46 years of age.

The detailed cohort description and follow-up protocol have been published previously [7, 8], and more information can also be found on the cohort’s webpage at https://www.oulu.fi/en/university/faculties-and-units/faculty-medicine/northern-finland-birth-cohorts-and-arctic-biobank/research-program-health-and-well-being. In 1997 (the 31-year follow-up), postal questionnaires regarding health, behaviour, work and social background were sent to all living cohort members with known addresses (*n* = 5608 women), and 4523 (81%) responded. In addition, those living in Northern Finland or in the Helsinki metropolitan area (*n* = 4074 women) were invited to a clinical examination; 3127 (77%) women participated. In 2012 (the 46-year follow-up), postal questionnaires and an invitation to a clinical examination were sent to all living cohort members with a known address (*n* = 5123 women). Among them, 3706 (72%) responded to the questionnaires, and 3280 (64%) participated in the clinical examination.

### Study outcome

The study outcome was the association of oligo/amenorrhea, hyperandrogenism, PCOS and menopause with detectable hs-TnT levels. Hs-TnT levels were measured at age 46, and results were available for 3146 women.

### Explanatory variables

#### Oligo/amenorrhea

For the follow-up at age 31, the postal questionnaire included questions regarding oligo/amenorrhea ‘Is your menstrual cycle often (more than twice a year) longer than 35 days?’ The response options were ‘Yes’, ‘No’ and ‘My periods are completely absent’. Women who responded ‘Yes’ or ‘My periods are completely absent’ were classified as having oligo/amenorrhea (*n* = 610), and their data were statistically compared to those of women with normal menstrual cycles – that is, those who responded ‘No’ to the above question (*n* = 3754).

#### Hyperandrogenism

The postal questionnaire at age 31 included the question, ‘Do you have bothersome, excessive body hair growth?’ Women who responded ‘Yes’ were considered to have clinical hyperandrogenism. Moreover, the presence of biochemical hyperandrogenism was defined as having elevated serum testosterone (*T* > 2.3 nmol/L) or the free androgen index (FAI > 5.6). Women with hyperandrogenism (clinical, biochemical or both, *n* = 795) were statistically compared to women without any signs of hyperandrogenism (*n* = 3654).

#### Polycystic ovary syndrome

PCOS was defined based on the Rotterdam criteria as involving at least two of the following features: oligo/amenorrhea, hyperandrogenism and/or anti-Müllerian hormone (AMH) ≥ 3.2 ng/mL (as a surrogate for ultrasonographic polycystic ovarian morphology) (*n* = 395) [9]. Women with PCOS were compared to women without oligo/amenorrhea or hyperandrogenism and with AMH < 3.2 ng/mL at age 31 (*n* = 1518). The formation of the PCOS group has previously been described in detail [9] and the approach of using AMH instead of ultrasonographic polycystic ovarian morphology in cases otherwise fulfilling only one PCOS feature follows the 2023 PCOS Guideline [10]. Of note, we have previously shown that women with PCOS in this cohort display typical anthropometric, hormonal and metabolic changes of PCOS as well as smaller prevalence of pregnancies by age 31 when compared to control women [9]. In addition, previous studies from this birth cohort have reported that women with typical PCOS features have smaller family sizes as well as increased odds for hypertensive disorders of pregnancy and gestational diabetes, although the associations were depending also on body mass index (BMI) [11–13].

#### Menopause and hormone replacement therapy

The follicle stimulating hormone (FSH) levels of 3161 women aged 46 were measured, and women with FSH over 25.0 IU/L were considered menopausal and compared to women with FSH below 25.0 IU/L [14]. Moreover, data on the use of hormonal replacement therapy (HRT) for menopause were gathered from the postal questionnaire at age 46; cohort members reported the names of all medications that they were taking along with the cause, dosage and frequency of use. Menopause based on FSH measurement, and the use of HRT were analysed separately, as not all menopausal women use HRT and HRT use may modify the effect of menopause on Hs-TnT.

#### BMI, blood pressure and left ventricular mass index

A trained professional measured the participants’ weights (kg) using a regularly calibrated digital scale and heights (cm) using a standard calibrated stadiometer. BMI was calculated (kg/m^2^) using the average of the two measurements of height and weight. A trained professional also measured brachial systolic and diastolic blood pressure (BP) three times at age 46 with a 1-min interval after 15 min of rest of the seated participants using an automated, oscillometric BP device (Omron Digital Automatic Blood Pressure Monitor Model M10-IT; Omron, Kyoto, Japan).

As a part of the clinical examinations at age 46, a subpopulation (*n* = 645 women) was enrolled for ­echocardiographic examination, the details of which have been previously published [15]. Transthoracic two-dimensional echocardiography was performed online by an experienced cardiologist (KK) using the General Electric Vivid E9 device with a M5S-D 1.5/4.6-MHz sector transducer for cardiovascular imaging (GE Health Medical, Horten, Norway). The American Society of Echocardiography guidelines were followed for all measurements, including evaluations of the left ventricular mass index (LVMI) [16].

#### Lifestyle habits

Multiple questions regarding the participants’ lifestyle habits, such as smoking and alcohol consumption, were included in the postal questionnaires. Based on the responses at age 46, respondents were classified as current smokers, former smokers for less than 6 months, former smokers for over 6 months and never smokers. Alcohol consumption was reported in grams per day (g/day) and classified as heavy use (>210 g/week), moderate use (150–210 g/week), light use (<150 g/week) or non-use.

### Laboratory methods

Blood samples were drawn in the morning, after overnight fasting. The laboratory analysis methods used to determine serum testosterone (T), sex-hormone binding globulin (SHBG), FAI, AMH, FSH, low-density lipoprotein (LDL) and fasting plasma (fP) glucose levels have previously been described in detail [9].

Hs-TnT was measured using an Elecsys® Troponin T-high sensitivity assay (Cobas e411, Roche Diagnostics GmbH, Sandhofer Strasse 116, D-68305 Mannheim, Germany), which employs two monoclonal antibodies directed against human cardiac troponin T. The assay’s measuring range is 3–10,000 ng/L. Values below the limit of detection are reported as < 3 ng/L and values above the measuring range as > 10,000 ng/L. The assay’s upper reference limit (99th percentile) for hs-TnT is 14 ng/L [17]. The limit of detection is the lowest hs-TnT value that can be measured when progressively diluting the sample, and a criterion for a high-sensitivity assay is that at least 50% of the test subjects should have detectable values above this level [18].

### Statistical methods

As the hs-TnT data were skewed, the data are presented as median values with 25 and 75 percentiles, and the Mann–Whitney U test was used to investigate statistical significance. The median hs-TnT values within the normal range (3.0–13.99 ng/L) were compared between cases and controls. Detectable hs-TnT levels (≥3.0 ng/L) were divided into four quartiles for a more detailed distribution. Categorical data were analysed using cross-tabulation and Pearson’s *χ*^2^ test or Fisher’s exact test. If either of these tests showed statistical significance when analysing the distribution of detectable hs-TnT quartiles, the *z*-test (Bonferroni proportions) was applied to reveal which percentages differed significantly from each other. Moreover, a logistic regression analysis was applied using a binary outcome: undetectable hs-TnT (<3.0 ng/L) versus detectable hs-TnT (≥3.0 ng/L). The unadjusted results are reported in Model I. Since BMI and hs-TnT levels are independently associated [19] and the main source of hs-TnT is myocardium, the regression analyses were adjusted for BMI and LVMI (Models II and III). The regression analyses were also further adjusted for other potential confounding factors: systolic BP, LDL and fP-glucose levels (Model IV). The results were reported as odds ratios with 95% confidence intervals.

Statistical analyses were performed using IBM SPSS Statistics 25.0 (IBM SPSS Statistics for Windows, v 25.0, released 2017, IBM Corp., Armonk, NY). *p* < .05 was considered statistically significant.

### Ethical approval

The study followed the principles of the Declaration of Helsinki. The Ethics Committee of the Northern Ostrobothnia Hospital District approved the research (decision number 94/2011). Participation was voluntary, and all participants signed an informed consent form.

## Results

**Figure 1.**
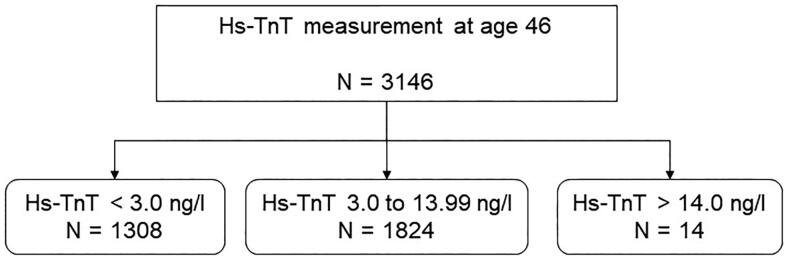
The distribution of undetectable (<3.0 ng/L), detectable (3.0–13.99 ng/L) and elevated (>14.0 ng/L) high-sensitivity troponin-T (Hs-TnT) in women aged 46.

Hs-TnT was detectable in 58% of the female population, below the detection limit in 41.6%, and above the normal limit in 0.4% (Figure 1). The median hs-TnT level in the female population was 4.00 [3.47; 4.74] ng/L.

### The association between female sex-specific health conditions and hs-TnT values at age 46

Table 1 shows the association between the selected health conditions and hs-TnT levels at age 46. Women with oligo/amenorrhea at age 31 had significantly higher hs-TnT levels at age 46 than women without oligo/amenorrhea. Menopausal women (FSH > 25 IU/L) had significantly higher hs-TnT levels than women with FSH < 25 IU/L, whereas the hs-TnT levels of HRT users and non-users did not differ. Women with PCOS or hyperandrogenism had hs-TnT levels comparable to those of the controls (Table 1).

**Table 1.** The association of lifestyle factors and medical conditions with hs-TnT values within the normal range at age 46.

Variables	Number of cases in each group	Hs-TnT level	*p* Value
Smoking			.082
Never	1061	4.03 [3.48; 4.79]	
Former > 6 months	389	3.89 [3.42; 4.57]	
Former < 6 months	28	3.85 [3.41; 4.51]	
Current	257	3.81 [3.45; 4.64]	
Alcohol consumption			**.001**
Non-use	212	4.19 [3.58; 5.05]	
Light use	1427	3.94 [3.46; 4.71]	
Moderate use	51	3.88 [3.37; 4.52]	
Heavy use	57	3.76 [3.32; 4.36]	
Oligo/amenorrhea			**.042**
No	1424	3.98 [3.44; 4.71]	
Yes	237	4.06 [3.59; 4.86]	
Hyperandrogenism			.751
No	1358	4.00 [3.44; 4.73]	
Yes	323	3.98 [3.52; 4.64]	
PCOS			.865
No	526	3.98 [3.42; 4.78]	
Yes	154	3.92 [3.57; 4.60]	
Menopause			**.015**
No	1473	3.95 [3.45; 4.68]	
Yes	327	4.15 [3.54; 4.91]	
Current use of HRT			.188
No	1777	3.98 [3.46; 4.71]	
Yes	47	4.27 [3.54; 5.05]	

HRT; hormonal replacement therapy for menopausal symptoms.

The difference between groups was analysed using the Mann–Whitney U test or the Kruskal–Wallis test. Oligo/amenorrhea, hyperandrogenism, and PCOS were screened at age 31, and the others at age 46. Statistically significant *p* values are in bold.

### The association between lifestyle habits and hs-TnT values at age 46

Hs-TnT levels varied according to the participants’ current alcohol consumption levels. Median hs-TnT levels decreased when alcohol consumption increased (Table 1). Smoking habits did not influence hs-TnT values (Table 1).

### Distribution of detectable hs-TnT quartiles

**Figure 2.**
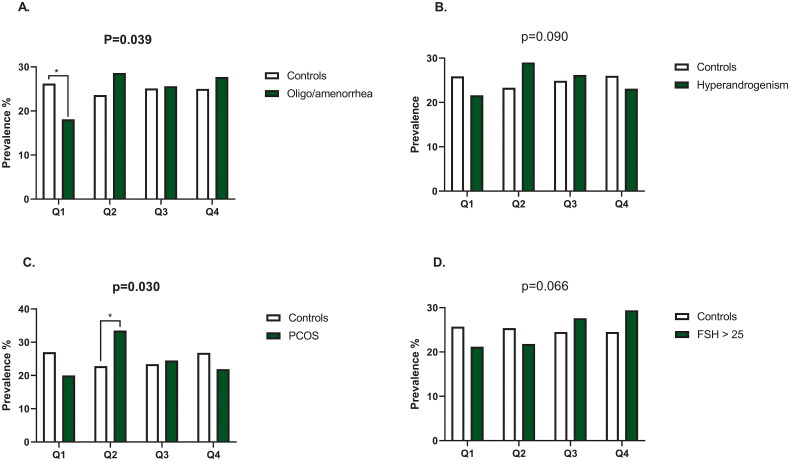
The prevalence of quartiles of detectable hs-TnT in women with oligo/amenorrhea (A), hyperandrogenism (B), polycystic ovary syndrome (C) and menopause (D). Q, quartile, PCOS, polycystic ovary syndrome, FSH, follicle stimulating hormone. The overall difference in distribution quartiles was analysed using Pearson’s *χ*^2^ test or Fisher’s exact test; statistically significant *p* values are in bold. If either of these tests showed statistical significance when analysing the distribution of detectable hs-TnT quartiles, the *z*-test (Bonferroni proportions) was applied to reveal which percentages differed significantly from each other. Only the statistically significant *z*-test results are reported (*p value < .05).

As shown in Figure 2, the distribution of detectable hs-TnT quartiles differed in women with PCOS and in women with oligo/amenorrhea, compared to the controls (*p* = .030 and *p* = .039 respectively). A significantly smaller proportion of women with oligo/amenorrhea were in the first quartile of detectable hs-TnT compared to women without oligo/amenorrhea (18.1% [*n* = 43] vs 26.2% [*n* = 376], *p* < .050). In addition, a significantly higher proportion of women with PCOS were in the second detectable quartile compared to the controls (33.5% [*n* = 52] vs 22.8% [*n* = 121], *p* < .050). The quartile distribution did not significantly differ in women with hyperandrogenism or menopause (Figure 2).

### Logistic regression analysis for detectable hs-TnT

In the logistic regression analysis, oligo/amenorrhea, hyperandrogenism, PCOS, menopause and current HRT use were not significantly associated with detectable hs-TnT levels (Table 2).

**Table 2.** Logistic regression analysis for detectable hs-TnT (>3.0 ng/L) at age 46.

Variables	Model I	Model II	Model III	Model IV
Oligo/amenorrhea	1.14 (0.91–1.41)	1.11 (0.89–1.38)	1.46 (0.87–2.47)	1.52 (0.90–2.57)
Hyperandrogenism	1.17 (0.96–1.42)	1.14 (0.94–1.38)	1.19 (0.75–1.89)	1.20 (0.75–1.92)
PCOS	1.05 (0.79–1.39)	1.03 (0.78–1.37)	1.33 (0.73–2.42)	1.51 (0.81–2.84)
Menopause	1.07 (0.90–1.31)	1.10 (0.91–1.33)	1.05 (0.64–1.73)	1.05 (0.63–1.74)
Current use of HRT	0.82 (0.53–1.29)	0.83 (0.53–1.29)	1.54 (0.46–5.21)	1.56 (0.46–5.32)
Smoking				
Never smoker (Ref.)	1.00	1.00	1.00	1.00
Former smoker > 6 months	1.01 (0.94–1.22)	0.99 (0.82–1.19)	0.92 (0.61–1.38)	0.94 (0.31–1.43)
Former smoker < 6 months	0.68 (0.40–1.17)	0.66 (0.38–1.14)	0.11 (0.01–1.00)	0.12 (0.01–1.03)
Current smoker	0.53 (0.44–0.65)	0.52 (0.43–0.63)	0.39 (0.24–0.62)	0.41 (0.25–0.66)
Alcohol consumption				
Non-use (Ref.)	1.00	1.00	1.00	1.00
Light use	0.79 (0.62–0.99)	0.79 (0.63–1.00)	0.62 (0.35–1.08)	0.65 (0.37–1.14)
Moderate use	0.80 (0.50–1.29)	0.80 (0.50–1.29)	1.03 (0.34–3.09)	1.05 (0.35–3.18)
Heavy use	0.64 (0.41–0.99)	0.64 (0.41–0.99)	0.57 (0.21–1.60)	0.64 (0.22–1.84)

PCOS, polycystic ovary syndrome; HRT, hormone replacement therapy.

The results are shown as odds ratios with 95% confidence intervals.

Model I: Crude model.

Model II: Body mass index (BMI) adjusted model.

Model III: BMI and left ventricle mass index (LVMI) adjusted model.

Model IV: Adjusted for BMI, LVMI, systolic blood pressure, low-density lipoprotein and fasting plasma glucose levels.

Compared to those who never smoked, current smoking associated inversely with detectable hs-TnT levels, whereas former smoking (both less than and over 6 months) was not significantly associated with detectable hs-TnT levels. Current smoking remained inversely associated with detectable hs-TnT levels even after adjusting for BMI, LVMI, systolic BP, LDL cholesterol and fP-glucose levels (Table 2).

Compared to the non-use of alcohol, light use and heavy use were associated with lower hs-TnT levels, whereas moderate use was not significantly associated with detectable hs-TnT (Model I). In the BMI-adjusted analysis, heavy use remained associated with lower detectable hs-TnT levels (Model II). However, in the models also adjusted for LVMI (Model III), as well as further for systolic BP, LDL cholesterol and fP-glucose levels (Model IV) heavy use of alcohol was no longer significantly associated with detectable hs-TnT.

## Discussion

The main finding of the present study was that women with oligo/amenorrhea and menopause had significantly higher hs-TnT levels than the controls. However, the actual difference was small, so the clinical significance may be limited. In the logistic regression analysis, none of the tested female sex-specific health conditions were significantly associated with detectable hs-TnT levels at age 46. Notably, alcohol consumption and current smoking habits were inversely associated with detectable hs-TnT levels.

This study was the first to examine the association between oligo/amenorrhea and hs-TnT levels. Women with oligo/amenorrhea at age 31 had significantly higher hs-TnT levels, and a smaller proportion of women with oligo/amenorrhea were in the lowest quartile of detectable hs-TnT, compared to women with normal menstrual cycles. Slightly elevated hs-TnT levels and the shift into higher quartiles of detectable hs-TnT could reflect an increased risk of CVD, as women with irregular menstrual cycles or amenorrhea have been reported to have an increased risk of premature CVD mortality [3]. Of note, in the present study, there were no significant differences in cardiovascular mortality between women with normal menstrual cycles and oligo/amenorrhea (data were not shown to ensure data protection and study participant’s anonymity). However, our study population might be too young to detect any differences in mortality.

Women with hyperandrogenism and those without hyperandrogenism had similar levels of hs-TnT, and hyperandrogenism was not associated with detectable hs-TnT in the logistic regression analysis. These findings are somewhat opposite to our hypothesis; we expected hyperandrogenism to be associated with hs-TnT levels, as hyperandrogenism is a known risk factor for CVD, at least in postmenopausal women [5]. We analysed testosterone levels using the gold-standard liquid chromatography-mass spectrometry method, which can detect low testosterone concentrations in women, to strengthen the reliability of our findings. However, the relationship between androgens and CVD could be complex, and more detailed androgen panels were unavailable in the study data.

Women with and without PCOS had similar hs-TnT levels, and PCOS was not associated with detectable hs-TnT levels in the regression analysis. However, a significantly higher proportion of women with PCOS were in the second quartile of detectable hs-TnT compared to women without PCOS. This might reflect an increased risk of CVD in women with PCOS, as evidenced by previous studies [4, 15]. We have recently reported that the same women with PCOS who were examined in the present study had a significantly higher risk for major cardiovascular events than control women by age 53 [20]. However, longer follow-up studies are needed to understand how CVD event risk develops at more advanced age, as women with PCOS may enter menopause later than their non-PCOS counterparts [21]. The possible later age at menopause in women with PCOS might be related to greater ovarian reserve, higher oestrogen levels or more effective DNA repair mechanism compared to women without PCOS, but the issue warrants further research.

Menopausal women had slightly higher hs-TnT levels than non-menopausal women. It should be noted that 46 years is a relatively early menopause age; thus, the population represented women with early ovarian failure and potentially high CVD morbidity. However, the logistic regression analysis showed that menopausal status was not significantly associated with detectable hs-TnT. In a previous cross-sectional analysis of the Dallas Heart Study, hs-TnT was not associated with menopausal status in the regression analysis [6], in line with our findings. Moreover, no previous studies have investigated the effects of postmenopausal HRT use on cardiac troponin levels, even though it is known to modify cardiovascular outcomes [22]. Our findings showed that users and nonusers of HRT had comparable hs-TnT levels. However, we were unable to investigate the effects of the administration route, timing, duration, and dose of HRT on hs-TnT levels due to the low number of women using HRT in the present study, warranting larger future studies.

Current smoking inversely associated with detectable hs-TnT levels even in the fully adjusted regression analysis (Model IV). In addition, the median hs-TnT levels decreased in the order of never smokers > former smokers for over six months > former smokers for less than 6 months > current smokers, but this was not statistically significant. These findings are in line with a previous study’s report that current smokers had significantly lower hs-TnI levels than never smokers or former smokers and that current smoking was inversely associated with hs-TnI levels [23]. The present study adds to the literature by confirming the inverse association between smoking and hs-TnT levels in a female-only population. Smoking is a well-established risk factor for CVD, so the inverse association between smoking and hs-TnT levels seems paradoxical. It has been speculated that current smokers have less left ventricular mass and more myocardial fibrosis than non-smokers and never smokers, which could explain the lower hs-TnT levels [17]. In testing this hypothesis, we found that adjusting the regression analysis for BMI and left ventricular mass index did not change the inverse association. It might be that certain unknown factors and their inverse causality can explain this paradoxical finding, as not all studies have found this inverse association [24].

Alcohol consumption was also inversely associated with detectable hs-TnT levels in the present study, but this association disappeared after adjusting for BMI and left ventricular mass (Model III). The Atherosclerosis Risk in Communities study found that persons who consumed 2–7 drinks per week were less likely than never drinkers to have increased hs-TnT (≥14 ng/L), whereas former drinkers were more likely to have increased hs-TnT concentrations [25]. Our study was the first to investigate the confounding effects of left ventricular mass index and BMI in the abovementioned association, and to show that left ventricular mass index largely explains this association.

### Strengths and limitations

The main strength of the present study was its population-based setup, as this reduced the risk of selection bias. Moreover, we were able to examine the effects of BMI and, for the first time to our knowledge, of left ventricular mass. The main limitation was that some of the analysed subgroups might have been underpowered. Further, menopausal status was based on a single FHS measurement, although the women classified as menopausal did present more climacteric symptoms (data not shown). Moreover, we lacked the pregnancy history data of cohort members until 46 years and were thus unable to test if pregnancy history associates with hs-TnT levels. In addition, even though hs-TnT is widely used as a biomarker of cardiac injury, it can sometimes originate from the skeletal muscles of patients with chronic skeletal muscle diseases [26].

## Conclusion

The present study was the first to investigate how oligo/amenorrhea, hyperandrogenism, PCOS, menopause and HRT use are associated with hs-TnT levels, thus contributing important and novel knowledge to the literature. Even though women with oligo/amenorrhea and menopause had significantly higher levels of hs-TnT compared to women without these conditions, the difference was small. Larger studies are required to better understand these complex associations, especially the effect of oligo/amenorrhea on cardiac troponin levels and overall cardiovascular health.

## Data Availability

NFBC data can be obtained from the University of Oulu, Infrastructure for Population Studies. Permission to use the data for research purposes can be requested *via* an electronic material request portal. When using the data, we followed the EU General Data Protection Regulation (679/2016) and the Finnish Data Protection Act. The use of personal data was based on the cohort participants’ written informed consent in their latest follow-up study, thus limiting its use. Please contact the NFBC project centre (NFBCprojectcenter@oulu.fi) or visit the cohort website (www.oulu.fi/nfbc) for more information.
